# An updated reference genome of *Barbatula barbatula* (Linnaeus, 1758)

**DOI:** 10.1038/s41597-025-04469-z

**Published:** 2025-01-22

**Authors:** Levente Laczkó, Nikoletta Andrea Nagy, Ágnes Nagy, Ágnes Maroda, Péter Sály

**Affiliations:** 1 One Health Institute, University of Debrecen, Debrecen, Hungary; 2 HUN-REN–UD Conservation Biology Research Group, University of Debrecen, Debrecen, Hungary; 3 Institute of Metagenomics, University of Debrecen, Debrecen, Hungary; 4 Department of Evolutionary Zoology and Human Biology, Faculty of Science andTechnology, University of Debrecen, Debrecen, Hungary; 5 HUN-REN–UD Behavioural Ecology Research Group, University of Debrecen, Debrecen, Hungary; 6 Hungarian Defence Forces Medical Centre, Budapest, Hungary; 7 MATE Department of Zoology and Ecology, Hungarian University of Agriculture and Life Sciences, Gödöllő, Hungary; 8 HUN-REN Institite of Aquatic Ecology, Centre for Ecological Research, Budapest, Hungary; 9 HUN-REN National Laboratory for Water Science and Water Security, Institute of Aquatic Ecology, Centre for Ecological Research, 29 Karolina Road, Budapest, H-1113 Hungary

**Keywords:** Genome, Biodiversity

## Abstract

The stone loach *Barbatula barbatula* is a benthic fish species widely distributed throughout Europe, primarily inhabiting stony upper sections of stream networks. This study presents an updated genome assembly of *B. barbatula*, contributing to the species’ available genomic resources for downstream applications such as conservation genetics. The draft assembly was 550 Mbp in size, with an N50 of 11.21 Mbp. We used the species’ available chromosome scaffolds to finish the genome. The final assembly had a BUSCO score of 96.7%. We identified 23270 protein-coding genes, and the proteome exhibited high completeness with BUSCO (93.1%) and OMArk (90.81%). Despite using multiple approaches to reduce duplicate contigs, we observed a relatively high duplicate ratio of 6.1% (BUSCO) and 8.52% (OMArk) in the annotations. We aimed to find microsatellite loci present in both the species’ publicly available genome and the new assembly to aid marker development for downstream analyses. This dataset serves as a reference for genomic analysis and is useful for developing markers to study the species’ biodiversity and support conservation efforts.

## Background & Summary

**Fig. 1:**
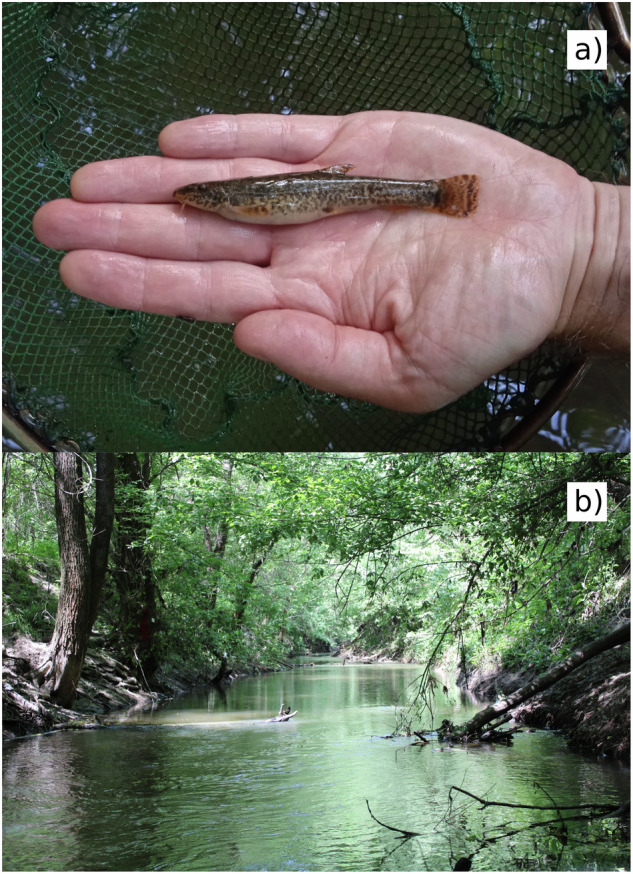
Stone loach *Barbatula barbatula* (Linnaeus, 1758) from the upper catchment of the Tarna river, Parádfürdő, Hungary (**a**). Photo taken by P.S. and Á.M. Tarna river in Tarnaszentmária, Hungary. One of the typical habitats of the stone loach, a gravel-bottom stream within a hilly and woody landscape (**b**). Photo taken by P.S. and Á.M.

The stone loach *Barbatula barbatula* (Linnaeus, 1758) (Cypriniformes, Nemacheilidae) is widely distributed throughout Europe and typically lives in stony, gravelly bottom sections of small to medium-sized streams with heterogeneous channel morphology and flow conditions. Its body shape is elongated, the head is slightly flattened dorso-ventrally, the cross-section of the trunk is roughly circular and the caudal peduncle is flattened laterally (Fig. 1). The caudal fin is slightly emarginate or truncate. It grows up to 160 mm long, but usually remains under 120 mm. Their most common size is 60–80 mm. The stone loach is a bottom-dwelling (benthic) species that feeds on small invertebrates^1^. It is native to the Danube river basin. Its status on the IUCN Red List is “Least Concern”^2^. The species is protected by Hungarian legislation for conservation.

Although the distribution area (i.e., range) of the species covers a large part of Europe, the occurrences are scattered, as the species mainly lives in the upper sections of stream networks (Strahler stream order 2 and 3). As lower river sections can function as ecological barriers (or non-preferred matrix habitats) for the rheofil brook-dwelling fish species^3,4^, dispersal between the stone loach populations living in geographically adjacent sub-catchments of large rivers is less likely, especially in homogeneously connected sub-catchments^5^. As a result, many local populations may be partially or completely genetically isolated despite the hydrological connectivity of the stream network^6^. It is hypothesized that stone loach populations living in adjacent streams exhibit high genetic variability (i.e., beta diversity) at the regional scale. Genetic variability could be even greater within a single stream if antropogenic barriers such as small dams and reservoirs separate populations. High-quality genome assemblies have had a significant impact on conservation genomics and provide important resources for the study of genetic diversity and evolutionary processes and traits of numerous organisms^7–12^. Genomic resources play a pivotal role in conservation genomics by providing comprehensive genetic resources for biodiversity research by providing markers that help to study genetic clusters at high resolution^e.g.,^^13^. Consequently, high-quality chromosome-level genome assemblies can be particularly useful for revealing genetic relationships between populations of neighboring stream catchments (regional spatial extent) and populations within a single stream (small-scale spatial extent). This not only helps to understand phylogeographic relationships at a fine scale, but also the dynamics of metapopulations and the effects of anthropogenic habitat changes on ecological connectivity. Therefore, information on the level and spatial distribution of the genetic diversity of the stone loach can support effective conservation management of the species. At the time of writing, only one genome assembly of the species is available, which has a relatively high ratio of missing genes (see below), which may hinder further exploitation of the resource. Multiple genome assemblies of the species may also be important for understanding the unique aspects of the species’ biology, including genome plasticity, identification of marker genes, and application of comparative genomics methods. With the advent of new sequencing technologies, microsatellites (SSRs) seem to remain useful markers due to their high variability, versatility and ubiquity in eukaryotic genomes, making them valuable for population genetic analyzes. Despite the advantages of a high number of SNP markers^14^, SSRs still appear to be a useful marker type to separate genetic clusters in conjunction with high-throughput sequencing methods that require only a minute amount of DNA^15^, and show high correlation with SNP markers in estimating genetic diversity and differentiation^16^. To date, ten microsatellite loci of the stone loach genome^17,18^ have been published and used for genotyping. However, the addition of further microsatellite markers isolated from a high-quality reference genome could significantly improve the resolution and scope of studies on genetic differentiation^19^, which could make an important contribution to the advancement of research targeting fine-scale patterns. Therefore, in this study we have paid particular attention to identifying potential microsatellite loci in the genome to aid future conservation genetic studies, for which microsatellites have been shown to be particularly useful^17,20–23^.

Here we report an updated assembly of *B. barbatula*, with which we aim to contribute to the available genomic resources of this species that could be used in downstream applications, particularly in conservation genetics. For the *de novo* assembly, we used third-generation sequencing reads generated with an Oxford Nanopore GridION sequencer, combining long and ultra-long reads with an overall 55-fold coverage. We estimated the genome size to be 588.40 Mbp. We used multiple assemblers and merged the draft assemblies to achieve higher genome contiguity (number of contigs = 286, N50 = 11.21 Mbp) (Table 1.). For the chromosome scaffolding, we used the publicly available whole genome sequence of the species, after which the final assembly comprised 25 chromosome scaffolds and 43 unplaced contigs with a total size of 550 Mbp. The chromosome scaffolds had a BUSCO score of 96.7% and the predicted proteome showed a completeness of 93.1% (Fig. 4.), both higher than the completeness score of the publicly available reference with a much lower ratio of unknown characters, demonstrating that Nanopore sequencing data alone can be used to reconstruct high quality genome sequences. We predicted 23270 protein-coding genes, of which 42.66% were involved in biological processes (BP), 29.55% in cell component formation (CC) and 27.78% in molecular functions (MF) (Fig. 3). The more complete and well-characterized genome of the species may be used in downstream analyzes, especially in conservation genetics, by serving as a reference for genomic analyzes and suitable for the development of markers that contribute to the assessment and monitoring of the genetic diversity of the species.

**Table 1:** Assessment of contiguity and completeness as estimated by QUAST 5.2.0^36^ and BUSCO 5.4.7^35^ in genome mode after different stages of the assembly process and polishing the assemblies with racon 1.5.0^29^ and medaka 1.11.3^30^.

Assembly	Shasta	nextDenovo	Merged	Merged and reduced	Decontaminated	Chromosome scaffold	Public reference (GCA_947034865.1)^43^
# contigs (> = 0 bp)	1392	430	324	289	286	68	100
# contigs (> = 1000 bp)	1269	430	324	289	286	68	100
# contigs (> = 5000 bp)	1052	430	324	289	286	68	90
# contigs (> = 10000 bp)	869	429	324	289	286	68	87
# contigs (> = 25000 bp)	630	421	316	286	283	65	42
# contigs (> = 50000 bp)	489	409	305	278	276	60	31
Total length (> = 0 bp)	504377085	552481893	552976402	550183806	549927078	549948878	617663352
Total length (> = 1000 bp)	504324218	552481893	552976402	550183806	549927078	549948878	617663352
Total length (> = 5000 bp)	503748801	552481893	552976402	550183806	549927078	549948878	617649352
Total length (> = 10000 bp)	502377626	552472087	552976402	550183806	549927078	549948878	617623753
Total length (> = 25000 bp)	498486426	552320729	552828901	550135342	549878614	549900414	616834017
Total length (> = 50000 bp)	493544348	551860728	552408854	549819351	549594396	549681916	616491988
# contigs	1134	430	324	289	286	68	100
Largest contig	12516413	16829151	22481428	22476465	22476465	32033418	36424156
Total length	504065283	552481893	552976402	550183806	549927078	549948878	617663352
GC (%)	39.47	39.58	39.56	39.55	39.55	39.55	39.69
N50	2247057	6119110	11211096	11211737	11211737	22548167	24423978
N90	428068	658227	1177969	1208022	1208022	18008182	21267045
auN	3120240.7	6721814.5	9445184.4	9488261.1	9492640	22387769.3	25231642.4
L50	59	28	20	20	20	11	12
L90	244	134	87	85	85	22	22
# N’s per 100 kbp	0	0	0	0	0	3.96	26.71
# N’s (% of total length)	0	0	0	0	0	21800 (0.004)	165000 (0.027)
Complete (%)	3340 (91.7)	3514 (96.5)	3506 (96.3)	3510 (96.5)	3519 (96.6)	3520 (96.7)	3501 (96.2)
Single copy (%)	3291 (90.4)	3458 (95.0)	3404 (93.5)	3409 (93.7)	3419 (93.9)	3426 (94.1)	3451 (94.8)
Duplicated (%)	49 (1.3)	56 (1.5)	102 (2.8)	101 (2.8)	100 (2.7)	94 (2.6)	50 (1.4)
Fragmented (%)	39 (1.1)	42 (1.2)	43 (1.2)	41 (1.1)	31 (0.9)	31 (0.9)	21 (0.6)
Missing (%)	261 (7.2)	84 (2.3)	91 (2.5)	89 (2.4)	90 (2.5)	89 (2.4)	118 (3.2)
Total number of BUSCOs	3640	3640	3640	3640	3640	3640	3640

## Methods

### Field collection of the specimens

Two adult specimens of the stone loach were caught by wading electrofishing (HansGrassl IG-200/2B, PDC, max. 10 kW per pulse) in a small river, the Tarna, Hungary, Central Europe. The length of the Tarna is 105 km and its catchment area is 2116 km^2,24^. The Tarna flows into the Zagyva, which has a confluence with the Tisza, the longest tributary of the Danube. The two specimens were caught at two sampling sites, where the Tarna has the size of a third-order stream.

The first specimen was collected on 2 September 2021 near the village of Kápolna, Hungary (lat: 47.76464711N, lon: 20.24230612E), and about one third of the caudal fin was clipped and taken as a tissue sample. Before clipping, the scissors were bathed in 70% ethanol for 10 minutes for disinfection, and the incision wound was treated with methylthioninium chloride (methylene blue). After clipping, the fish was placed in a bucket of river water with methylene blue added for approximately 20 minutes to recover and was finally released back into its habitat. The second specimen was collected on 22 July 2022 near the village of Tarnaszentmária, Hungary (lat: 47.87097883N, lon: 20.20819846E), which is about 13.2 km upstream from the sampling site in Kápolna. This specimen did not recover after the electroshock (i.e., it died), so the caudal half of its body was taken as a tissue sample. The tissue samples were brought to the laboratory in tubes filled with 96% ethanol and stored in the refrigerator at 4 °C until DNA isolation. The handling of wild fish was carried out with the authorization of the respective authorities (permits HaGF/125/2020 and HAGF/120/2022). The field collection was supervised by a hydrobiological desk officer from the competent National Park Directorate.

### DNA isolation and sequencing

We isolated total genomic DNA from 20 mg of the caudal fin of both specimens using a DNeasy Blood and Tissue Kit (Qiagen, Hilden, Germany) following the manufacturer’s protocol for Purification of Total DNA from Animal Tissues. We then prepared five sequencing libraries using the Ligation Sequencing Kit SQK-LSK110 (Oxford Nanopore Technologies, Oxford, UK) according to the manufacturer’s protocol for Genomic DNA by Ligation. In addition, we prepared high molecular weight genomic DNA isolates for ultra-long-read sequencing from 37 mg muscle tissue of the second specimen by using the Monarch HMW DNA Extraction Kit for Tissue (New England Biolabs, Ipswich, UK) with some modifications. In brief, we homogenized the fresh muscle tissue sample using a mortar and pestle and then added 600 μl of HMW gDNA Tissue Lysis Buffer and 20 μl of Proteinase K. We incubated the homogenate at 56 °C for 45 minutes with agitating at 700 rpm, then added 10 μl RNAse A to remove the RNA content, and further incubated the homogenate at the same setting. We added 300 μl of Protein Separation Solution and centrifuged the mixture at 16000 × *g* at 4 °C for 20 min to remove the proteins from the solution. We then added large glass beads to the separated upper layer and precipitated the DNA with 550 μl isopropanol. We used 500 μl gDNA Wash Buffer to purify the isolates and eluted the genomic DNA in 760 μl EEB buffer from the Ultra-long DNA Sequencing Kit (Oxford Nanopore Technologies, Oxford, UK). We proceeded with library preparation following the manufacturer’s protocol of the Ultra-long DNA Sequencing Kit SQK-ULK110 (Oxford Nanopore Technologies, Oxford, UK).

We generated sequencing data using the Oxford Nanopore GridION platform by loading the six libraries onto five R9.4.1 flow cells (Oxford Nanopore Technologies, Oxford, UK). We used real-time super-high accuracy basecalling with MinKNOW 21.11.7 and Guppy 5.1.13 (Oxford Nanopore Technologies, Oxford, UK) to achieve the highest possible accuracy rate. The sequencing runs generated a total of 34.98 Gb of raw genomic data in five batches. Long-read sequencing of four batches yielded 12.99 Gb with an N50 of 12.57 kb, 10.21 Gb with an N50 of 9.51 kb, 9.35 Gb with an N50 of 18.81 kb and 1.86 Gb with an N50 of 15.36 kb. The sequencing of the library prepared with SQK-ULK110 resulted in 576 Mb sequencing data with a read N50 value of 13.16 kb.

### Read quality filtering and preprocessing

We pooled all sequencing reads and filtered them with NanoLyse to remove the DNA control strand, and then used NanoFilt to exclude reads shorter than 500 base pairs (bp) or with a mean quality score of less than seven. To ensure that the sequencing data was free of sequencing adapters, we trimmed 50 bp from both ends of the reads. After filtering, we retained 3699636 reads with a read length N50 of 14031, corresponding to a total of 34.512 Gbp. We then estimated the genome size of the species using CovEst 0.5.6^25^, that tolerates an error rate of up to 10% without significantly affecting the accuracy of the results. We prepared *k*-mer frequency histogram for this estimation with KMC 3.1.1^26^, using the pooled sequencing dataset (i.e., all sequencing reads of the six libraries) as input and setting the *k*-mer length to 21 and the upper coverage threshold to 10000. CovEst, with an estimated error rate of 0.02%, estimated the coverage to be 55.07-fold and the genome size to be 588.40 Mbp.

### Genome assembly

**Fig. 2:**
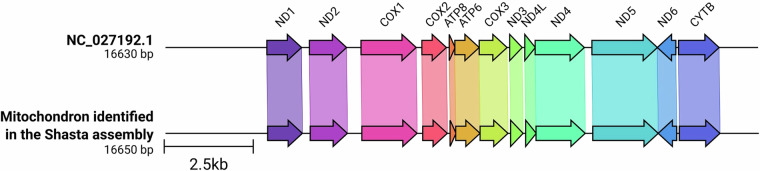
Structural comparison of the publicly available mitochondrial genome of *Barbatula barbatula* and the *de novo* assembled mitochondrial genome. The figure reconstructed using clinker 0.0.27^32^ was further edited with Inkscape 1.2.2^63^ to improve readability.

We assembled, scaffolded and polished the genome sequence in multiple steps. First, we assembled the long reads using Shasta 0.11.1^27^ and nextDenovo 2.5.2^28^. We then aligned the filtered long reads to both original assemblies and used racon 1.5.0^29^ and medaka 1.11.3^30^ with the r941_min_sup_g507 model to increase the accuracy of the assemblies. We checked the mitochondrial sequences in both assemblies using blastn 2.14.0 + using the available mitochondrial genome of the species (NC_027192.1) as query and the assemblies as subject sequences. The evaluation of these results revealed that the Shasta assembly contained the mitochondrion on a separate contig and appeared to be circular with a total length of 16650 bp, whereas the mitochondrion in the nextDenovo assembly appeared to be twice as long (31756 bp) and contained the mitochondrial sequence in two identical copies, one of which was inverted and formed a loop. We accepted the polished mitochondrial sequence of the Shasta assembly as representative of the species and annotated the mitochondrial genome using the MITOS2 web server^31^. To verify the accuracy of the mitochondrial assembly, we compared its structure to the publicly available mitochondrial genome using clinker^32^, for which we curated the *de novo* assembled mitochondrion to have the same start site as the reference (Fig. 2.). We identified the same number of genes as in the reference (NC_027192.1) in the same order and orientation. Before proceeding with additional experiments to improve contiguity and completeness, we excluded mitochondrial sequences from both initial assemblies.

We merged all 1392 contigs of the Shasta (N50 = 2.25 Mbp) and 430 contigs of the nextDenovo assemblies (N50 = 6.12 Mbp) using quickmerge 0.3^33^, where we specified the nextDenovo as the first assembly and the Shasta as the second assembly, resulting in a more continuous genome assembly of 324 contigs (N50 = 11.21 Mbp) (Table 1). We then polished the assembly with racon 1.5.0^29^ and medaka 1.11.3^30^ as described above. We used seqkit 2.8.0^34^ to assess contiguity and ran BUSCO 5.4.7^35^ in genome mode to assess completeness after each major step of the assembly process and summarized the contiguity statistics with QUAST 5.2.0^36^. Since we observed a larger ratio of BUSCO duplicates after merging the assemblies (Table 1.), we attempted to resolve false duplications using pseudohaploid 7c01418^37^ by running create_pseudohaploid.sh. Additionally, we used redundans 0.11^38^ with the options–nogapclosing and–noscaffolding. We tested several parameter combinations for the ratios of identity (0.80, 0.85, 0.90, 0.95, 1.0) and overlap (0.80, 0.85, 0.90, 0.95, 1.0) to detect duplicate contigs and estimated the optimal identity to be 1.0 and the optimal overlap to be 0.80, which combination resulted in the fewest contigs without reducing the ratio of complete BUSCO genes. Before and after each attempt to detect duplicates, we performed genome polishing as previously described. The reduced assembly consisted of 289 contigs with a total size of 550.18 Mbp and an N50 of 112.12 Mbp and an improved BUSCO score after polishing (Table 1).

The presence of contamination is common in eukaryotic genome assemblies and should be removed prior to downstream analyzes^39,40^, which is often carried out based on the taxonomic classification of the contigs. We used BERTax 0.1^41^, an accurate taxonomic classifier to identify contaminants, and excluded all contigs that were not classified as Chordata (n = 3, 256.73 kbp). To improve the accuracy of identification, we also used blastn 2.14.0 + to verify the identity of contigs that were not classified as a species of Pisces (genera *Carassius*, *Clupea*, *Cottoperca*, *Denticeps*, *Erpetoichthys*, *Gadus*, *Myripristis*, *Podarcis* or *Scleropages*) using the complete NCBI nucleotide BLAST database as the reference. The most probable hits (n >  = 5) for all contigs that were not classified as fish species by BERTax (genera *Methanocella*, *Podarcis*, *Schistosoma* or unknown) belonged to a member of Pisces in all cases; therefore, we accepted them as representative contigs of the target genome. After decontamination, we polished the assembly again in the same way as after the previous main steps and then used RagTag 2.1.0^42^ to scaffold the contigs. For the reference chromosome scaffold, we used the available reference genome of *Barbatula barbatula* (GCA_947034865.1^43^), which chromosome model was reconstructed using Hi-C sequencing^43^. RagTag successfully scaffoled 243 of 286 contigs (545.66 Mbp) with 218 gap regions (218 kbp) on 25 chromosomes, leaving 43 contigs (4.26 Mbp) unplaced. Finally, we polished the scaffolds again using racon^29^ and medaka^30^ with the previously applied settings, that yielded the final assembly with much fewer unknown characters (3.96/100 kbp) than those in the available reference genome (26.72/100 kbp) of the species (Table 1.). Contamination removal and chromosome scaffolding both improved the BUSCO score by 0.1%, resulting in a final BUSCO score of 96.7%, with 2.6% of genes duplicated and 2.4% missing, a higher overall score and a higher ratio of duplicated genes than in the available reference genome, but also 0.8% fewer missing genes (Table 1, Fig. 4a).

### Genome annotation

Prior to gene prediction and functional annotation, we soft-masked both the genomic contigs and the scaffolded chromosomes using Red 2.0^44^, which identified 527037 repeat regions in the contigs with a minimum length of 14 bp and a maximum length of 136966 bp (total length 178.16 Mbp, mean = 338.03 bp), accounting for 63.56% of the assembly. The chromosome scaffolds contained 527880 repeat regions with a maximum length of 135423 bp (total length = 178.66 Mbp, mean = 338.45 bp), representing 32.48% of the total genome length. We then annotated rRNAs with barrnap 0.9^45^ and tRNAs with ARAGORGN 1.2.38^46^. We used BRAKER 3.0.8^47,48^ to predict the sequence, location and structure of protein-coding genes by a combination of *ab initio* and evidence-based prediction. We performed *ab initio* prediction with Augustus 3.5.0^49^. For homology-based prediction, we used the Vertebrata_odb11 reference database (https://bioinf.uni-greifswald.de/bioinf/partitioned_odb11/Vertebrata.fa.gz) and generated hints with ProtHint 2.6.0^50^, then generated a training set for Augustus using these hints with GeneMark-EP 4.71_lic^50^. We used the agat_sp_merge_annotations.pl script of Another Gtf/Gff Analysis Toolkit 1.4.2 (AGAT^51^) to merge the *ab initio* and homology-based annotations, then the agat_sp_keep_longest_isoform.pl script to filter for only the longest products per gene. We achieved the amino acid sequences of the gene products with the agat_sp_extract_sequences.pl script of AGAT. We submitted these sequences to the PANNZER web server^52^ (http://ekhidna2.biocenter.helsinki.fi/sanspanz/) to predict the function of each putative gene.

**Fig. 3:**
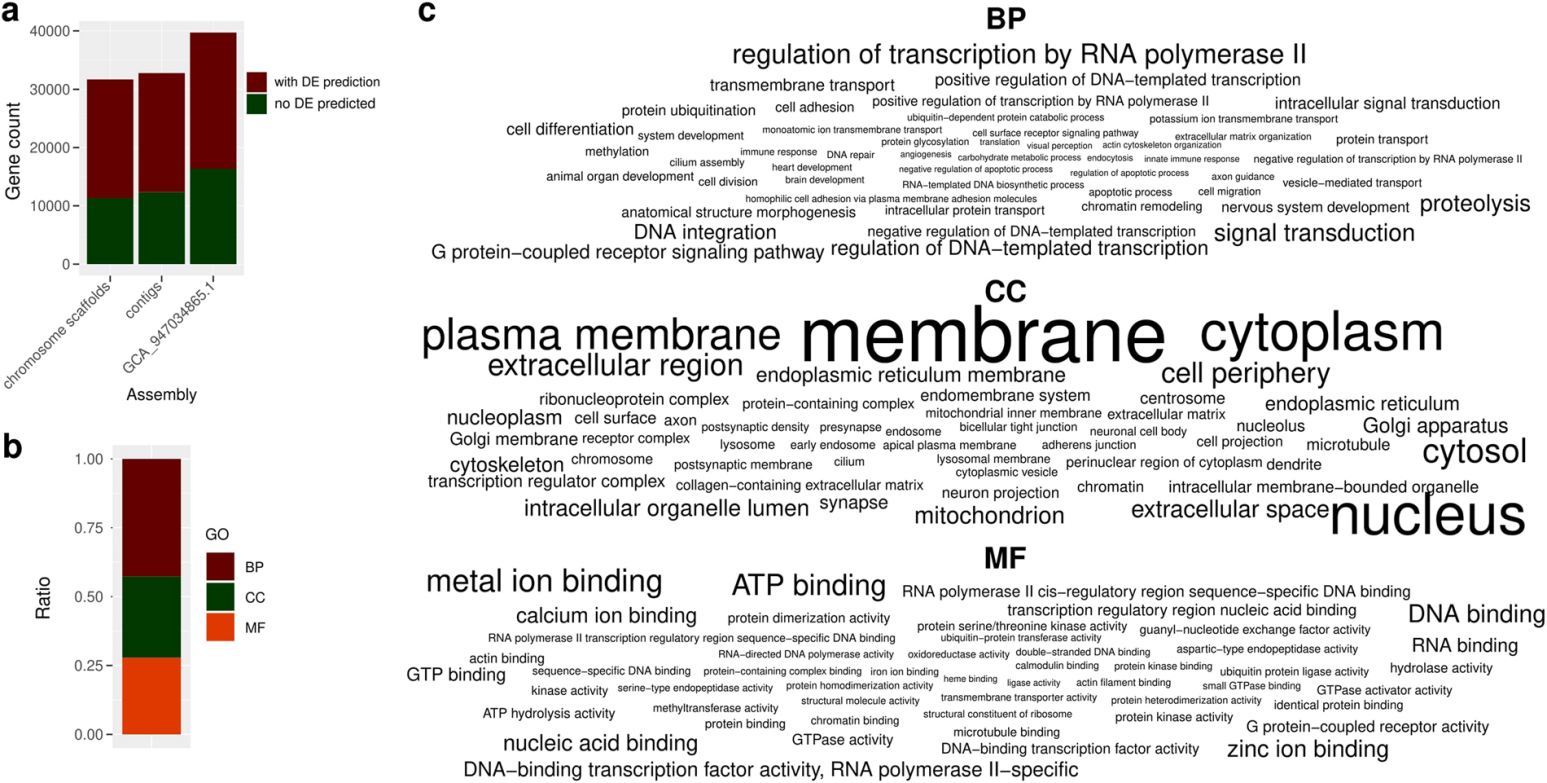
Functional annotation of the predicted genes in the chromosome scaffolds. The figure shows the total number of predicted genes in the newly assembled contigs, chromosome scaffolds and reference genome (GCA_947034865.1)^43^ as a bar chart stacked to represent the ratio of genes that received a GO term after functional annotation. (**a**) The ratio of GO terms belonging to different ontologies is shown as a bar chart (**b**), and the 50 most frequent functions found in each ontology (biological process – BP, cell composition – CC, molecular function – MF) are shown as a word cloud (**c**), where the font size is proportional to the frequency of each function in the annotation.

In this way, we identified 32763 putative genes in the contigs and 31679 in the chromosome scaffolds, less than in the available reference (n = 39710). In the newly assembled genome, a bigger proportion of the predicted genes could be functionally annotated (20983 in the contigs and 23270 in the scaffolds, 64.04–93.46% of the putative genes) than in the public reference (GCA_947034865.1)^43^ (23954, 60.32% of structurally predicted genes) (Fig. 3a).

We identified 42.66% of the genes as being involved in biological processes (BP), with regulation of transcription by RNA polymerase II, signal transduction, proteolysis, DNA integration and regulation of DNA-templated transcription being the most abundant functions. 29.55% of the genes were assigned to a GO term belonging to the cell composition (CC) ontology, and most of them played a role in forming membranes, nucleus, cytoplasm, plasma membrane and cytosol. All the remaining genes (27.78%) had a molecular function (MF), and metal ion binding, ATP binding, DNA binding, zinc ion and nucleic acid binding appeared to be the most common gene functions (Fig. 3b,c).

**Fig. 4:**
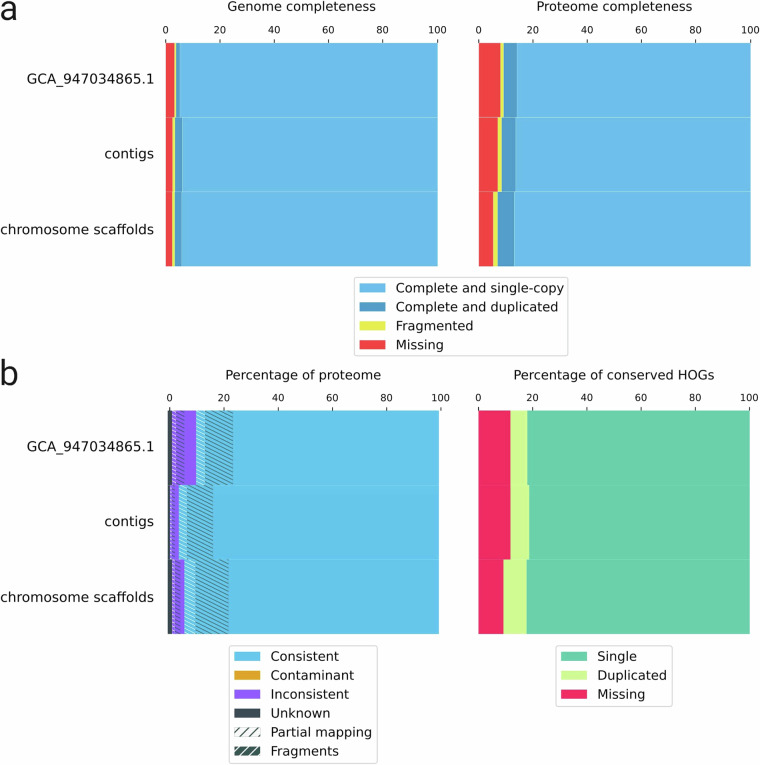
Genome and annotation completeness as assessed by BUSCO (**a**) and OMArk (**b**).

To ensure the high quality of annotations, we ran BUSCO 5.4.7^35^ in proteome mode against the actinopterygii_odb10 database, which predicted higher completeness of both contigs (91.6%) and scaffolds (93.1%) than that of the reference genome (90.9%). The assemblies presented here also had a higher duplication score (5.2% in the contigs and 6.1% in the chromosomes) than the publicly available reference (5.0%), but were missing only 7.0% and 5.3% BUSCOs, respectively, less than the missing fraction of the publicly available assemblies (8.0%; Fig. 4a). Additionally, we ran OMArk 0.3.0 (https://omark.omabrowser.org/home/ release 2024.06)^53^ using OMAmer 2.0.3 (database: Jul2023). This analysis predicted a lower proportion of missing genes in the chromosomes (9.19%) of the new version of the genome than in our contig-level assembly (11.79%) and in the publicly available assembly (11.77%), but similar to the BUSCO analysis, found a higher ratio of duplicates (6.9% in the contigs and 8.52% in the chromosomes) in the assemblies presented here than in the publicly available genome (6.24%) (Fig. 4b). Additionally, the ratio of consistently placed genes were higher in our assemblies (95.89% and 93.85%) than in the public version (89.51%) (Fig. 4b). OMArk did not detect contamination in either assembly and identified them all as members of the Otophysi, with more than 98% of the predicted genes associated with this lineage.

### Identification of SSRs

We screened SSR loci in both the publicly available genome assembly and the assembly presented in this study to find variable loci that are present in both assemblies and thus can be used directly in downstream applications. First, we ran MISA 2.1^54^ using both the publicly available genome and the chromosome scaffolds as separate input genomes with unit size definitions of 1–10 2–6 3–5 4–5 5-5 6-5, setting interruptions in compound SSRs to 100 bp, and requiring .gff output. MISA identified a total of 182496 SSR loci in assembly GCA_947034865.1^43^, of which 28371 appeared to be monomeric, 134631 dinucleotides, 8865 trinucleotides, 6897 tetranucleotide repeats, and 3732 microsatellite feature repeats. In the chromosome scaffolds, MISA found 212862 SSR loci, of which 55672 were monomeric, 138279 dinucleotide, 8319 trinucleotide, 7176 tetranucleotide repeats, and 3416 microsatellite features. Next, we exported the flanking region of each locus from both genomes using bedtools getfasta 2.31.0^55^ with coordinates spanning 1000 bp before and after the SSR locus. We used BLAST 2.14.0^56^ to match the loci of the two assemblies, using SSRs from GCA_947034865.1^43^ as database and SSRs of the chromosome scaffolds as query. We kept only unique hits and accepted a locus if the BLAST hits were present on the same scaffold and the hits had higher coverage and identity than 90%. In this way, we identified 26209 common SSR loci, of which 283 appeared to be monomorphic^57^. The common loci consisted of 4115 monomeric, 19292 dinucleotide, 2076 trinucleotide and 647 tetranucleotide repeats, and 79 microsatellite features.

## Data Records

We deposited all data described in this study in the NCBI database under BioProject PRJNA1049631. The raw data can be found in the Sequence Read Archive (SRA) database under accessions SRR27127808^58^ and SRR27127809^59^, whereas the *Barbatula barbatula* genome assembly can be found in the Assembly database under accession GCA_037178815.1^60^. The assembly submitted to GeBank can be found under accession number JAXOFQ000000000^61^. The structural and functional annotation of the assembly as well as the contigs and chromosome scaffolds and identified microsatellites are made public in the Zenodo data repository under 10.5281/zenodo.1450203657.

## Technical Validation

We carefully filtered the sequencing dataset with NanoLyse and NanoFilt to remove the DNA control strand, sequencing adapters, and low-quality reads to ensure a relatively low error rate and increase assembly contiguity and completeness. We compared the structure of the mitochondrial genome with the most closely related mitochondrial reference genome using clinker to validate its structure and annotation. We polished all assemblies with racon and medaka before and after each step attempting to increase contiguity, and checked the quality of the assemblies for contiguity and completeness using QUAST and BUSCO. Additionally, we checked the completeness of the proteome using OMArk. We ensured that the final assembly was free of contamination by checking the taxonomic classification of the contigs prior to chromosome scaffolding using Bertax. We used *ab initio* and evidence-based gene predictions to obtain high quality genome annotation and assessed the number of functionally annotated genes and the number of gene duplications in a phylogenetic context.

## Data Availability

We did not use any custom code in this study. The version and parameters of the bioinformatic tools used in this study were described in the Methods section. If a parameter was used other than the default value, we described it accordingly.
